# Strengthening exercises improve knee muscle strength and performance but not pain in ACL‐reconstructed individuals: A systematic review and meta‐analysis of randomised controlled trials

**DOI:** 10.1002/jeo2.70576

**Published:** 2025-12-17

**Authors:** Mostafa Jalili Bafrouei, Fateme Khorramroo, Hooman Minoonejad, Seyed Hamed Mousavi

**Affiliations:** ^1^ Department of Sport Injuries and Biomechanics, Faculty of Sport Sciences and Health University of Tehran Tehran Iran

**Keywords:** ACL, eccentric, isokinetic, isotonic, resistance

## Abstract

**Purpose:**

Deficits in quadriceps and hamstring strength, functional performance and pain are common after anterior cruciate ligament reconstruction (ACLR). The role of strengthening exercises (SE) in addressing these outcomes remains uncertain. This systematic review and meta‐analysis aimed to evaluate the effects of SE on quadriceps and hamstring strength, functional performance and pain in individuals after primary ACLR.

**Methods:**

Four databases (PubMed, Web of Science, Scopus and Embase) were searched from inception to July 2025. Eligibility criteria included randomised controlled trials (RCTs) on adults with ACLR evaluating SE versus conventional rehabilitation. Data extraction followed PRISMA 2020 guidelines. The risk of bias was assessed using the Cochrane PEDro scale. Random‐effects models were used to calculate standardised mean differences (SMD) with 95% confidence intervals (CI). Heterogeneity was assessed with *I*², and subgroup/sensitivity analyses and publication bias were performed.

**Results:**

Nineteen RCTs (*n* = 818) were included. SE significantly improved quadriceps strength (SMD: 0.76; 95% CI: 0.46 to 1.05; *p* < 0.01; *I*² = 0%, *p* = 0.48) and hamstring strength (SMD: 0.70; 95% CI: 0.41 to 1.0; *p* < 0.01; *I*² = 0%, *p* = 0.43). Functional performance (single‐leg hop test, Lysholm score, ADL questionnaire, ROM extension) also improved with SE. However, knee pain outcomes showed no significant changes (SMD: −0.32; 95% CI: −0.98 to 0.34; *p* = 0.34; *I*² = 86%, *p* < 0.01).

**Conclusions:**

SE significantly enhances knee strength and functional performance in individuals with ACLR, although their effects on pain remain unclear. Clinically, incorporating SE into rehabilitation protocols is recommended to improve recovery outcomes, accelerate return to sport (RTS) and enhance readiness. Further studies are warranted to clarify their impact on pain management.

**Level of Evidence:**

Level I.

**Abbreviations**
ACL: anterior cruciate ligament
ACLR: anterior cruciate ligament reconstruction
ADL: activities of daily living
CI: confidence interval
CKRS: Cincinnati Knee Rating Score
FK: Fateme Khorramroo
HM: Hooman Minoonejad
IKDC: International Knee Documentation Committee
LSI: Limb Symmetry Index
MJB: Mostafa Jalili Bafrouei
MVIC: maximal voluntary isometric contraction
QOL: quality of life
SE: strength exercises
SHM: Seyed Hamed Mousavi
VAS: visual analogue scale
WOMAC: Western Ontario and McMaster Universities score

## INTRODUCTION

Anterior cruciate ligament (ACL) injuries are frequent in young, active individuals and often require surgical reconstruction to restore dynamic knee stability and functional capacity [5, 14, 29]. Despite advancements in surgical techniques, individuals undergoing ACLR frequently experience persistent neuromuscular deficits, including proprioceptive dysfunction, residual muscle weakness, altered kinematics and chronic pain that compromise knee joint stability and motor control [30].

Quadriceps and hamstring strength deficits are particularly critical, as they contribute to reduced knee stability, abnormal gait and increased risk of re‐injury [26]. Evidence indicates that each 1% improvement in quadriceps strength reduces re‐injury risk by approximately 3%, while imbalances in the hamstring‐to‐quadriceps ratio further increase susceptibility to secondary knee injuries, and the development of compensatory movement patterns may compromise joint stability [23]. Additionally, premature return to sport before 9 months post‐surgery is strongly associated with higher re‐injury rates, highlighting the importance of restoring muscle strength before resuming high‐level activity [10, 35, 39, 54].

SE, particularly those targeting the quadriceps, hamstrings and hip musculature, are fundamental to ACLR rehabilitation because they directly influence joint loading patterns, graft strain and movement control [54]. The quadriceps and hamstrings act as dynamic stabilisers that counter anterior tibial translation and rotational stresses on the reconstructed ligament, thereby reducing strain on the graft [6]. Moreover, hip abductor and external rotator strength contribute to controlling knee valgus and internal rotation during dynamic tasks, minimising abnormal joint moments and compensatory movement patterns [39]. From a neuromechanical perspective, progressive resistance training enhances motor unit recruitment, prevents muscle atrophy and improves proprioceptive and motor coordination, ultimately supporting superior functional outcomes [1, 3, 31, 47]. Moreover, SE can reduce pain and lower the risk of complications such as stiffness and early osteoarthritis [43]. Despite their established role as a cornerstone of ACLR rehabilitation, strengthening protocols differ substantially in contraction type (isometric, isotonic, or isokinetic), load progression, targeted muscle groups and duration across clinical settings, which may lead to heterogeneous recovery profiles after ACLR [13].

Given the variability in strengthening approaches across rehabilitation settings, understanding how these variables modulate neuromuscular recovery and functional readiness is essential to refining evidence‐based rehabilitation strategies after ACLR. Several studies have examined the effects of SE on strength, functional performance and pain in individuals after ACLR. However, findings remain inconsistent and sometimes contradictory, limiting the ability to draw definitive conclusions. Therefore, this systematic review and meta‐analysis aimed to determine not only whether SE improves knee muscle strength, functional performance and pain after ACLR, but also how variations in exercise type, load and duration influence neuromuscular recovery and readiness to RTS.

## METHODS

### Study design

This systematic review adhered to the Preferred Reporting Items for Systematic Reviews and Meta‐Analysis PRISMA guidelines [2]. The objective was to identify, evaluate and synthesise studies that examined knee strength, the functional outcomes and knee pain of the SE in individuals with ACLR. The protocol was registered in PROSPERO (CRD42024620787).

### Search strategy

Relevant studies were identified through a comprehensive search of four electronic databases: PubMed, Web of Science, Scopus and Embase. The search was conducted on 16 July 2025. The search strategy was based on broad terms and related synonyms organised, reported in Supporting Information: Table 1. Additionally, reference lists from previous systematic reviews on SE in ACLR populations were manually searched to ensure the inclusion of all relevant studies.

### Eligibility criteria

Studies were included based on the following criteria:

Population: participants with a history of ACLR, regardless of post‐ACLR time, age, or surgery type.

Intervention: any SE defined as a structured, progressive, resistance‐based intervention (e.g., isotonic, isokinetic, or elastic resistance) designed to improve knee strength, functional performance and pain, including exercises that target the knee muscles directly or indirectly through multi‐joint or lower‐limb strengthening approaches.

Comparators: any control intervention focused on range of motion, swelling reduction and low‐intensity activities (e.g., traditional exercise, rehabilitation, standard exercise, or physiotherapy standard).

Outcomes: primary outcomes included pain‐related measures such as VAS and KOOS‐pain. Secondary outcomes included objective and self‐reported assessments of knee strength and functional performance, including the single‐leg hop test, ROM extension, Lysholm score and ADL questionnaire.

Study design: randomised controlled trials (RCTs).

Language and publication status: peer‐reviewed publications in English.

### Study selection

All records identified through the search strategy were imported into the Comprehensive Meta‐Analysis software (CMA, version 7.4) and duplicates were removed. MJB and FK independently screened the titles and abstracts to identify relevant studies. The full texts of potentially eligible articles were then obtained and evaluated against the predefined inclusion criteria. Any disagreements between reviewers were resolved through consensus or by consulting two additional reviewers (HM, SHM), who were consulted to reach consensus.

For data extraction, MJB and FK collected information from the included studies using a standardised extraction form to ensure consistency. The following information was recorded: author and year, study design, participants’ demographics, tools, intervention and duration, outcome measures and funding information.

### Quality assessment

Methodological quality was appraised using the PEDro checklist [17]. Scores were interpreted as: poor (<4), fair (4–5), good (6–8) or excellent (9–10) [19]. Discrepancies were resolved by consensus. Publication bias was evaluated with Egger's test, with *p* > 0.05 indicating no significant funnel plot asymmetry.

### Data collection

MJB and FK extracted all data from the included studies, with HM and SHM verifying the accuracy of the extracted data. The data on strength, function and pain, such as Cincinnati Knee Rating Score (CKRS), Lysholm score, hop test scores and visual analogue scale (VAS), commonly used in the management of ACLR in clinical settings, were collected. Additionally, the number of sessions, study design, variables, intervention, number of participants and their characteristics (age, sex, height, mass) and tools were systematically extracted from the included studies and are reported in Supporting Information: Table 2.

### Synthesis of results

Standard mean difference (SMD) and 95% confidence intervals (CI) were calculated using a random‐effects model instead of a fixed‐effects model in CMA version 7.4 in meta‐analysis, due to expected heterogeneity in study designs, tools and outcome measures, which was performed when two or more studies evaluated the same outcome measure using similar methodologies. Statistical heterogeneity of the combined data was assessed using *I*² statistics and corresponding *p*‐values (*p* < 0.05). To assess potential publication bias, both the Funnel Plot and Egger's regression test were applied. In cases where evidence of publication bias was identified, the trim‐and‐fill method was employed to estimate its potential impact on the overall meta‐analytic outcomes and to approximate the number of potentially missing studies. The results were interpreted based on the levels of evidence established by Tulder et al. [19], as modified by Jalili Bafrouei, which provides guidelines for conducting and reporting systematic reviews in this research domain. Findings were organised by type of reported outcomes in Supporting Information: Table 3.

## RESULTS

### Study selection

**Figure 1:**
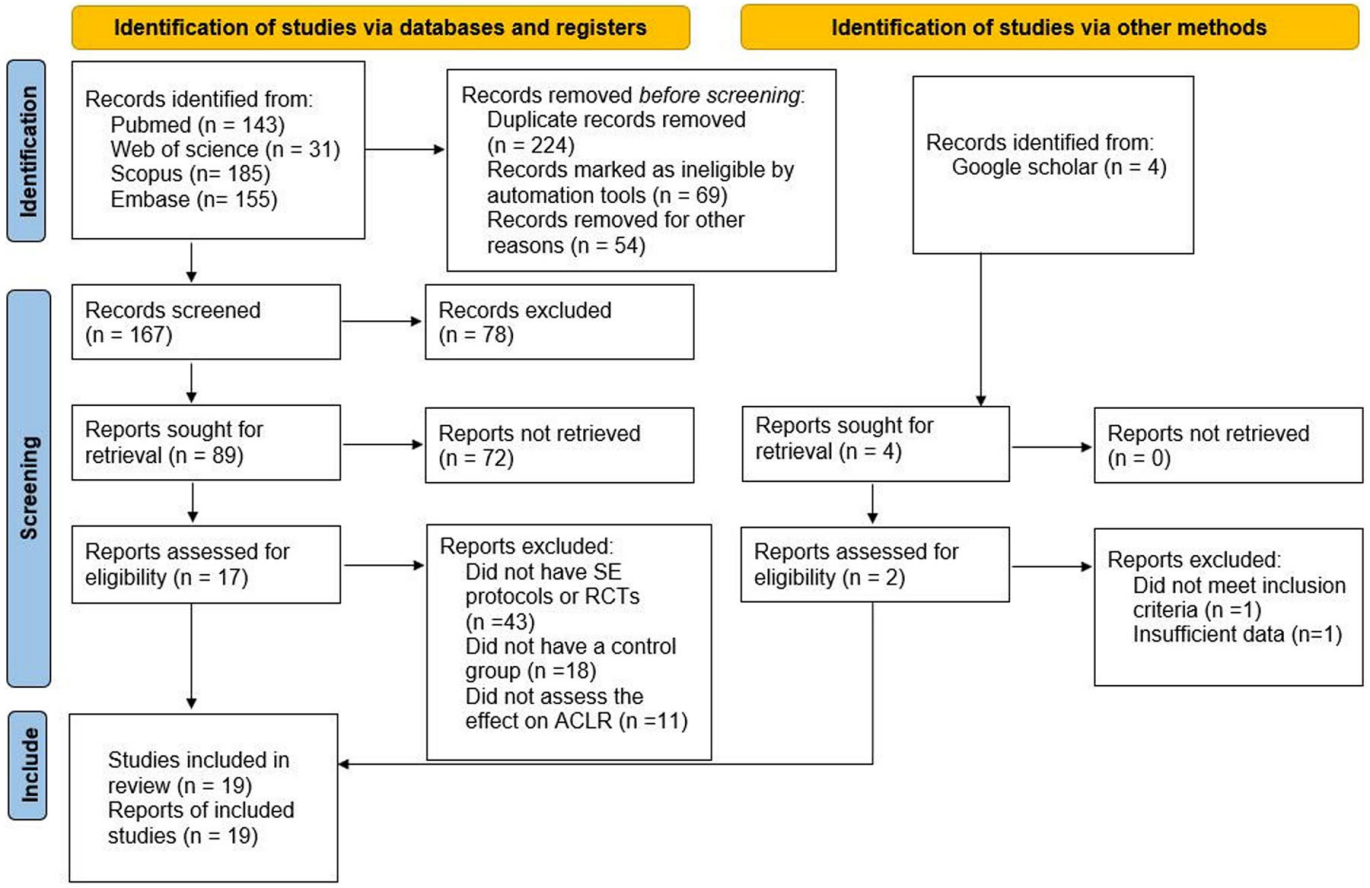
Flow chart of study selection process.

The main literature search yielded a total of 514 items (PubMed (143 studies), Web of Science (31), Scopus (185) and Embase (155)) from which 224 items remained after duplicate removal. After screening the titles and abstracts, 19 studies were included [8, 12, 17, 18, 20, 21, 32, 33, 34, 36, 37, 38, 39, 40, 42, 47, 50, 51, 53]. Figure 1 shows the flow diagram of the selection process and the number of excluded studies at each stage. Any missing or incomplete data in the included studies were addressed by contacting the corresponding authors when possible. If sufficient data were not provided, the study was excluded from the analysis. The primary exclusion criteria comprised: absence of established SE protocols (*n* = 43), lack of control group implementation (*n* = 18) and failure to evaluate ACLR outcomes (*n* = 11).

### Study characteristics

Publication years spanned from 2005 to 2023 (median: 2014), with 89.5% (17/19) of studies published after 2014. Collectively, the total sample size of the 19 included studies was 818. A total of eight studies exclusively enroled male participants [17, 18, 33, 36, 37, 38, 39, 40] one study focused solely on female participants [32] and 10 studies included a mixed‐gender sample comprising both males and females [8, 11, 20, 21, 34, 42, 47, 50, 51, 53]. Geographically, studies originated from the United States [8, 20, 21, 32, 33, 42], Germany [17, 18], the UK [38], Australia [47], Denmark [11], Serbia and Romania [50], Poland [39], Iran [36], South Africa [37], Brazil [51], Turkey [34], China [53] and Egypt [40]. All 19 studies [8, 12, 17, 18, 20, 21, 32, 33, 34, 36, 37, 38, 39, 40, 42, 47, 50, 51, 53] underwent rigorous quality assessment using standardised appraisal tools.

### Quality assessment

Supporting Information: Table 4 shows the results of the quality assessment using the PEDro scale, ranging from 5/10 to 10/10. The highest study, Moubarak et al. (2022), achieved an excellent rating (PEDro = 10/10). The lowest‐rated studies were Garrison et al. (2014) and Gerber et al. (2009), which received a fair rating (PEDro = 5/10). The methodological quality and risk of bias of included studies were independently assessed using the RoB‐2 scale [16] for randomised trials in Supporting Information: Table 5 [49].

### Outcome measured

Of the 19 studies [8, 12, 17, 18, 20, 21, 32, 33, 34, 36, 37, 38, 39, 40, 42, 47, 50, 51, 53], 16 targeted strength [8, 12, 17, 18, 21, 33, 34, 36, 37, 38, 40, 42, 47, 50, 51, 53], 14 measured performance [8, 12, 20, 21, 32, 33, 34, 36, 38, 39, 40, 47, 50, 51] and 5 studies targeted pain [12, 20, 36, 39, 47].

### Effects of SE on knee strength

**Figure 2:**
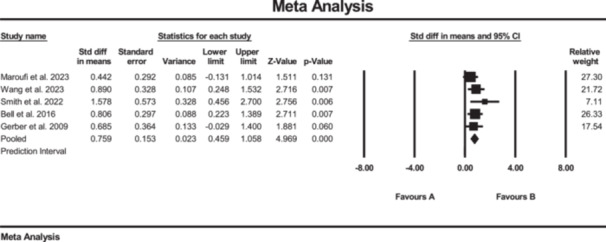
Meta‐analysis comparing quadriceps muscle strength (SMD: 0.76, SE: 0.15) between group B (the SE group, *N* = 87) and group A (the conventional rehabilitation group, *N* = 87).

Sixteen studies investigated the effect of SE on knee strength, measured by quadriceps muscle strength [8, 21, 36, 42, 53], hamstrings muscle strength [8, 21, 36, 42, 53], hip external rotation muscle strength [20, 36, 39, 47], hip abduction muscle strength [8, 36] and the isokinetic concentric and eccentric quadriceps muscle at 60°/s [37, 47]. The results of the meta‐analysis suggested strong evidence of significantly increased changes in quadriceps muscle strength (SMD: 0.76; 95% CI: 0.46 to 1.05; *p* < 0.01; *I*² = 0%, *p* = 0.48) in the SE group compared to the conventional rehabilitation group (Figure 2). For quadriceps muscle strength, the Egger's test did not reach statistical significance (*p* = 0.90); however, the visual inspection of the funnel plot suggested a possible asymmetry, indicating a potential risk of publication bias (see Supporting Information: File 6).

**Figure 3:**
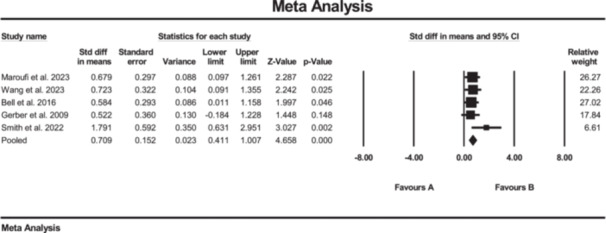
Meta‐analysis comparing the hamstrings muscle strength (SMD: 0.70, SE: 0.15) between group B (the SE group, *N* = 107) and group A (the conventional rehabilitation group, *N* = 85).

**Figure 4:**
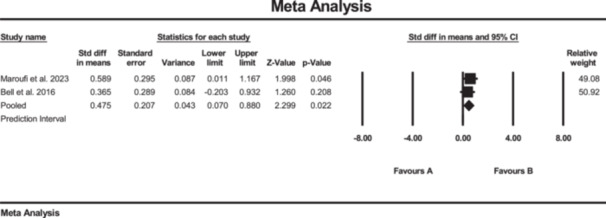
Meta‐analysis comparing hip external rotation muscle strength (SMD: 0.47, SE: 0.47) between group B (the SE group, *N* = 61) and group A (the conventional rehabilitation group, *N* = 42).

Additionally, the results of the meta‐analysis suggested strong evidence of significantly increased changes in hamstrings muscle strength (SMD: 0.70; 95% CI: 0.41 to 1.0; *p* < 0.01; *I*² = 0%, *p* = 0.43) in the SE group compared to the conventional rehabilitation group (Figure 3). Furthermore, no evidence of publication bias was detected based on the funnel plot and Egger's test, although the limited number of included studies may reduce the sensitivity of these assessments. Furthermore, the results of the meta‐analysis showed moderate evidence of a significant increase in hip external rotation muscle strength (SMD: 0.47; 95% CI: 0.07 to 0.88; *p* = 0.02; *I*² = 0%, *p* = 0.5) (Figure 4).

Moreover, the results of meta‐analysis showed moderate evidence of a lack of significant changes for hip abduction muscle strength (SMD: 0.35; 95% CI: −0.46 to 0.78; *p* < 0.83; *I*² = 0%, *p* = 0.99), isokinetic concentric quadriceps muscle at 60°/s (SMD: 0.06; 95% CI: −0.29 to 0.41; *p* = 0.73; I² = 0%, *p* = 0.90) and eccentric quadriceps muscle at 60°/s (SMD: 0.07; 95% CI: −0.28 to 0.42; *p* = 0.69; *I*² = 0%, *p* = 0.94) in the SE group compared to the conventional rehabilitation group (see Supporting Information: File 7).

### Effects of SE on performance

**Figure 5:**
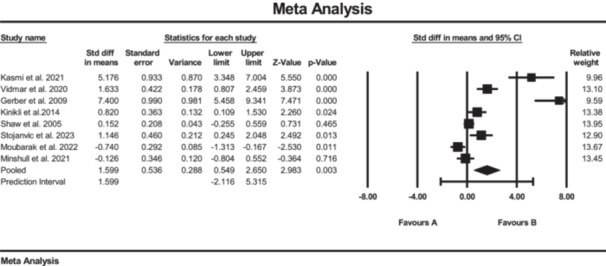
Meta‐analysis comparing the single‐leg hop test (SMD: 1.59, SE: 0.53) between group B (the SE group, *N* = 161) and group A (the conventional rehabilitation group, *N* = 153).

Fourteen studies investigated the effect of SE on performance, using the single‐leg hop test (SLHT) [21, 32, 34, 38, 39, 47, 50, 51], activities of daily living (ADL) questionnaire [11, 21, 47], Lysholm scale [21, 32, 34, 51], range of motion (ROM) in knee extension [20, 36, 39, 47], time up and go test (TUG) [36, 40], 6‐metre walk test (6MWT) [32, 36], triple hop test (THT) [32, 47, 50], International Knee Documentation Committee (IKDC) scale [8, 20, 33] and quality of life (QOL) questionnaire [11, 34, 36]. The results of the meta‐analysis suggested strong evidence of significant changes in the SLHT (SMD: 1.59; 95% CI: 0.55 to 2.65; *p* < 0.01; *I*² = 93%, *p* < 0.01) in the SE group compared to the conventional rehabilitation group (Figure 5). The Egger's test indicated significant asymmetry (*p* = 0.006) and visual inspection of the funnel plot also suggested the presence of publication bias (see Supporting Information: File 6).

**Figure 6:**
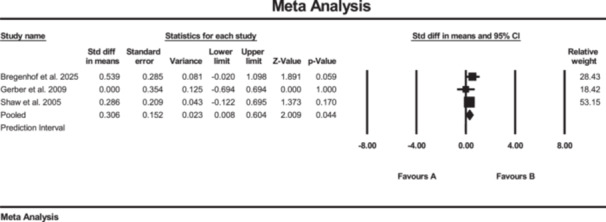
Meta‐analysis comparing the ADL questionnaire (SMD: 0.30, SE: 0.15) between group B (the SE group, *N* = 90) and group A (the conventional rehabilitation group, *N* = 86).

**Figure 7:**
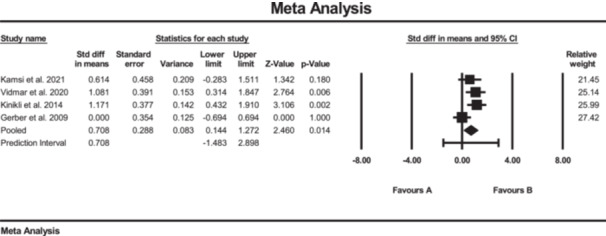
Meta‐analysis comparing the Lysholm scale (SMD: 0.70, SE: 0.28) between group B (the SE group, *N* = 58) and group A (the conventional rehabilitation group, *N* = 57).

**Figure 8:**
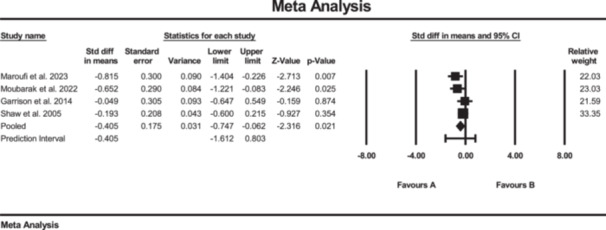
Meta‐analysis comparing ROM knee extension (SMD: −0.40, SE:0.17) between group B (the SE group, *N* = 128) and group A (the conventional rehabilitation group, *N* = 116).

The results of the meta‐analysis suggested strong evidence of significant changes in the ADL questionnaire (SMD: 0.30; 95% CI: 0.00 to 0.60; *p* = 0.04; *I*² = 0%, *p* = 0.49) (Figure 6), Lysholm scale (SMD: 0.70; 95% CI: 0.14 to 1.27; *p* < 0.01; *I*² = 53%, *p* = 0.09) (Figure 7) and ROM in knee extension (SMD: −0.40; 95% CI: −0.74 to −0.06; *p* = 0.02; *I*² = 39%, *p* = 0.17) (Figure 8) in the SE group compared to the conventional rehabilitation group. Furthermore, no evidence of publication bias was detected based on the funnel plot and Egger's test, although the limited number of included studies may reduce the sensitivity of these assessments.

Moderate evidence of significant changes in the TUG test (SMD: 0.83; 95% CI: 0.14 to 1.52; *p* < 0.01; *I*² = 51%, *p* = 0.15) in the SE group compared to the conventional rehabilitation group (see Supporting Information: File 7). Moreover, results of the meta‐analysis suggested conflicting evidence of changes in 6‐MWT (SMD: 1.05; 95% CI: −0.16 to 0.27; *p* = 0.09; *I*² = 76%, *p* = 0.03) and QOL questionnaire (SMD: 0.62; 95% CI: −0.22 to 1.46; *p* = 0.14; *I*² = 81%, *p* < 0.01) for the SE group compared to the conventional rehabilitation group (see Supporting Information: File 7). Furthermore, no evidence of publication bias was detected based on the funnel plot and Egger's test, although the limited number of included studies may reduce the sensitivity of these assessments.

Besides, the results of the meta‐analysis suggested conflicting evidence of changes in the THT (SMD: 1.41; 95% CI: −0.54 to 3.37; *p* = 0.15; *I*² = 93%, *p* < 0.01) and IKDC scale (SMD: 1.78; 95% CI: −0.09 to 3.68; *p* = 0.06; *I*² = 93%, *p* < 0.01) for the SE group compared to the conventional rehabilitation group (see Supporting Information: File 7). Although visual inspection of the funnel plots suggested potential publication bias for THT and IKDC, the Egger's test results were not statistically significant (*p* = 0.287 and *p* = 0.314, respectively), indicating that the observed asymmetry may not be conclusive (see Supporting Information: File 6).

### Effects of SE on pain

**Figure 9:**
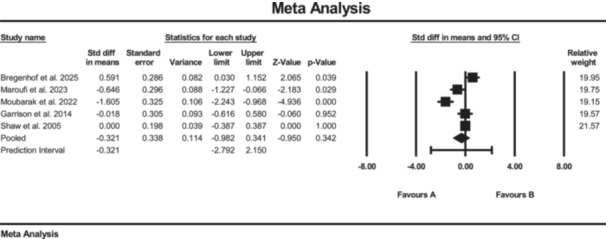
Meta‐analysis comparing pain (SMD: 0.32, SE: 0.33) between group B (the SE group, *N* = 143) and group A (the conventional rehabilitation group, *N* = 152).

Five studies investigated the effect of strength training on pain, measured by VAS [11, 20, 36, 39, 47]. The results of the meta‐analysis suggested conflicting evidence of changes in pain score (SMD: −0.32; 95% CI: −0.98 to 0.34; *p* = 0.34; *I*² = 86%, *p* < 0.01) for the SE group compared to the conventional rehabilitation group (Figure 9). Moreover, no evidence of publication bias was detected based on the funnel plot and Egger's test, although the limited number of included studies may reduce the sensitivity of these assessments.

## DISCUSSION

This systematic review and meta‐analysis demonstrated that SE significantly improves quadriceps and hamstring strength, as well as functional performance outcomes such as the SLHT, Lysholm score and ADL in individuals post‐ACLR. However, SE did not consistently reduce knee pain. These findings highlight that while muscle strength and function improve with SE, achieving full readiness for RTS remains multifactorial and requires more comprehensive neuromuscular retraining.

### Effects of SE on knee muscle strength

The meta‐analysis suggested strong evidence of improvements in muscle strength between the SE and conventional rehabilitation groups. These findings suggest that SE can enhance the muscle strength of the knee post‐ACLR, leading to more efficient dynamic movement patterns during activities and quality of life [22]. Improving knee flexor and extensor strength is crucial for reducing the risk of further injuries, such as ligament tears [41]. Importance of rapid strength recovery for safe return to sport, with isokinetic training proving beneficial in enhancing both extensor and flexor muscle strength post‐ACL reconstruction [52]. Additionally, SE enhances muscle activation and movement efficiency, which are essential for dynamic tasks. This improvement is particularly evident in exercises that involve unstable conditions, which challenge the body's ability to stabilise and coordinate movements [48, 55]. Specifically, maintaining postural stability during performance is critical for individuals recovering from ACLR, where knee instability and muscle imbalance are prevalent [56].

However, not all strengthening outcomes were significantly improved. Some studies reported a lack of improvement in tight abduction muscle strength [8, 36] and the isokinetic concentric and eccentric quadriceps muscle at 60°/s [37, 47], which may indicate that SE alone may not be sufficient to restore full joint stability or correct all deficits and require high integration of post‐ACLR due to neuromuscular adaptation [4]. Additionally, extending rehabilitation timelines to 10‐12 months yielded greater strength gains compared to traditional 6‐month protocols [22], underscoring the benefits of prolonged and progressive SE regimens, due to fear of re‐injury and immaturity of graft tissue [22, 39]. The observed improvements in muscle strength could be attributed to enhanced knee control and better integration of proximal and distal muscle function of the lower limb, as muscle strength plays a key role in recovering knee post‐ACLR and optimising lower‐limb mobility [47]. Yet, more research is needed to establish the precise mechanisms through which SE influences strength factors, particularly for knee‐specific outcomes.

### Effects of SE on performance

The meta‐analysis provided moderate evidence supporting a significant improvement in performance following SE in individuals with ACLR. Sixteen studies evaluated the impact of SE on performance through various functional tests. This improvement is attributed to increased muscle strength and power, which are critical for the explosive movements required in the hop test. For instance, a study found that starting with knee exercises improved functional activities more than performance, while starting with hip exercises improved performance more than functional activities [25]. Additionally, quadriceps strength and reactive strength index were significant predictors of single‐leg hop performance, indicating that both muscle strength and plyometric characteristics are important [9]. Another study highlighted that quadriceps strength was positively associated with better hop distance and landing mechanics post‐rehabilitation [28].

This improvement is likely due to SE's positive effects on balance, postural stability, activation and movement, which optimise the coordination and efficiency of lower extremity movements during dynamic tasks [15, 57]. Moreover, muscle strength training, which involves high‐velocity contractions, might be more effective than traditional strength training in improving ADL performance [27], ROM in knee extension [36] and the Lysholm score [46].

However, some studies, particularly those using the THT and IKDC, did not report significant improvements following SE in ACLR individuals. This lack of improvement in the THT performance may be linked to fear of re‐injury during landing, as previous injury history is a well‐established risk factor for re‐injury [7, 15, 39]. Furthermore, the IKDC is influenced by various factors, including the surgical technique used, rehabilitation duration [16], muscle strength around the knee [15], psychological factors and pain perception [47].

### Effects of SE on pain

The meta‐analysis suggested findings were inconsistent across studies regarding pain reduction by VAS between the SE and conventional rehabilitation groups. On one hand, some studies suggest that SE may not directly alleviate pain, particularly during the early stages of rehabilitation, when inflammation, swelling and surgical side effects are prevalent [15, 20, 47].

On the other hand, other studies indicate that pain alleviation may require more time [44]. The variability in pain outcomes could be influenced by factors such as the type and intensity of SE, the type of graft used in ACLR and the timing of the intervention within the rehabilitation process [39, 45]. It is important to recognise that pain relief following ACLR is multifactorial, involving both physiological and psychological elements [57]. While SE primarily aims to improve muscle balance, strength ratios and functional capacity [16], it may not directly address inflammation or the mechanical strain on the knee joint resulting from ACL deficiency [24]. Therefore, focusing solely on strengthening exercises may not fully resolve the underlying sources of knee pain or inflammation associated with ACLR. Given the conflicting results regarding the effects of SE on individuals with ACLR, it is recommended that further studies be conducted on this issue.

### Limitations and recommendations for future research

Several limitations should be considered when interpreting the findings on SE. Many studies included small sample sizes and short intervention durations, which restricts the generalisability and long‐term applicability of the results. Although the pooled analysis indicated 0% heterogeneity, this value should be interpreted cautiously. Such a result likely reflects the limited number of available studies rather than true consistency across trials, and it may mask underlying variability in participants, interventions, or outcome measures. The timing of interventions post‐ACLR may also influence outcomes, as early‐stage exercises may not provide sufficient stimulus for significant improvements in strength or performance. Furthermore, variations in exercise protocols (e.g., intensity, progression, targeted muscle groups) and study designs (e.g., conventional rehabilitation groups, outcome measures) complicate the ability to draw clear and definitive conclusions. Moreover, the type of surgery, whether autograft or allograft and the specific muscle tendon used for the procedure can significantly impact rehabilitation outcomes. These factors should be separately and effectively assessed to strengthen training. These discrepancies highlight the need for more rigorous, standardised research to better evaluate the role of SE in ACLR rehabilitation. Additionally, most studies have predominantly focused on male athletes, limiting the generalisability of the findings to female athletes and non‐athletic populations. Future research should aim to conduct larger, well‐controlled trials with extended follow‐up periods, standardised protocols and comprehensive biomechanical assessments, while addressing the current limitations.

## CONCLUSIONS

SE effectively restores knee muscle strength and functional performance after ACLR; however, their effects on pain relief remain inconsistent. Successful RTS requires a combination of muscular, neuromuscular and psychological recovery. Progressive SE, administered by clinicians and physiotherapists and integrated with proprioceptive, plyometric and cognitive components, represents the most evidence‐based approach to achieving complete functional restoration and reducing the risk of re‐injury.

## AUTHOR CONTRIBUTIONS

S.H.M. contributed to conceptualisation, methodology, software development, validation, investigation, data curation, drafting the original manuscript, reviewing and editing, supervision and project administration. M.J.B. contributed to conceptualisation, software development, methodology, validation, formal analysis, investigation, data curation, drafting the original manuscript, reviewing and editing, supervision and project administration. F.K. contributed to conceptualisation, validation, formal analysis, investigation and data curation. H.M. contributed to conceptualisation, validation, methodology, reviewing and editing, data curation, formal analysis and investigation.

## CONFLICT OF INTEREST STATEMENT

The authors declare no conflicts of interest.

## ETHICS STATEMENT

None declared.

## Supporting information

Table 1. Key terms and Boolean operators.

Table 2. Study characteristics for the included studies.

Table 3. Definitions of modified level of evidence by Jalili Bafrouei.

Table 4. The results of the PEDro quality assessment scale in RCTs.

Table 5. Risk of bias in RCTs.

Supplementary File 6.

Supplementary File 7.

Supplementary File 8.

PRISMA_2020_checklist.

## Data Availability

All relevant data are included in the article.
