# Supplementary material for: Strengthening exercises improve knee muscle strength and performance but not pain in ACL‐reconstructed individuals: A systematic review and meta‐analysis of randomised controlled trials
Source: J Exp Orthop. 2025 Dec 17;12(4):e70576. doi: 10.1002/jeo2.70576 (PMC12709656; doi:10.1002/jeo2.70576)
Supplement: Supplementary file 1 — Table 1. Key terms and Boolean operators. [file JEO2-12-e70576-s009.docx]

Table 1. Key terms and Boolean operators

| Database | Key terms and Boolean operators |
| --- | --- |
| PubMed | Title/Abstract: (1) ("anterior cruciate ligament reconstruction" OR “ACL reconstruction” OR ACLR OR ACL-R OR "ACL surgery"), Title/Abstract: (2) (“strength* exercise*” OR “strength* train*” OR "progressive eccentric" OR “eccentric train*” OR "early strength*" OR "isometric exercise*" OR "isometric train*" OR ”eccentric exercise*” OR “concentric train*” OR “hip strength*” OR “quadriceps exercise*”), Title/Abstract: (3) (strength* OR function* OR pain OR performance OR power), All fields: (4) 1 AND 2 AND 3. |
| Web of Science | Topic search: (“anterior cruciate ligament reconstruction” OR “ACL reconstruction” OR ACLR OR ACL-R OR “ACL surgery”) AND Topic search: (“strength* exercise*” OR “strength* train*” OR "progressive eccentric" OR “eccentric train*” OR "early strength*" OR "isometric exercise*" OR "isometric train*" OR ”eccentric exercise*” OR “concentric train*” OR “hip strength*” OR “quadriceps exercise*”) AND Topic search: (strength* OR function* OR pain OR performance OR power). |
| Scopus | Title/Abstract/Keywords: (“anterior cruciate ligament reconstruction” OR “ACL reconstruction” OR ACLR OR ACL-R OR “ACL surgery”) AND Title/Abstract/Keywords: (“strength* exercise*” OR “strength* train*” OR "progressive eccentric" OR “eccentric train*” OR "early strength*" OR "isometric exercise*" OR "isometric train*" OR ”eccentric exercise*” OR “concentric train*” OR “hip strength*” OR “quadriceps exercise*”) AND Title/Abstract/Keywords: (strength* OR function* OR pain OR performance OR power). |
| Embase | Title/Abstract/Keywords: (“anterior cruciate ligament reconstruction” OR “ACL reconstruction” OR ACLR OR ACL-R OR “ACL surgery”) AND Title/Abstract/Keywords: (“strength* exercise*” OR “strength* train*” OR "progressive eccentric" OR “eccentric train*” OR "early strength*" OR "isometric exercise*" OR "isometric train*" OR ”eccentric exercise*” OR “concentric train*” OR “hip strength*” OR “quadriceps exercise*”) AND Title/Abstract/Keywords: (strength* OR function* OR pain OR performance OR power). |
