# Supplementary material for: Strengthening exercises improve knee muscle strength and performance but not pain in ACL‐reconstructed individuals: A systematic review and meta‐analysis of randomised controlled trials
Source: J Exp Orthop. 2025 Dec 17;12(4):e70576. doi: 10.1002/jeo2.70576 (PMC12709656; doi:10.1002/jeo2.70576)
Supplement: Supplementary file 2 — Table 2. Study characteristics for the included studies. [file JEO2-12-e70576-s001.docx]

Table 2. Study characteristics for the included studies.

| **Authors** | **Study design** | **Samples** | **Duration** | **Outcomes** | **Tools** | **Time post-ACLR** | **Sample size and sex** | **Age (years)** | **Weight (kg)** | **Height (cm)** | **BMI (kg/m2)** | **Type of grafts** |
| --- | --- | --- | --- | --- | --- | --- | --- | --- | --- | --- | --- | --- |
| **Bregenhof et al. 2023** | **RCT** | **ACLR** | **12 Weeks** | **Maximal isometric knee flexor and extensor strength, hamstring-to-quadriceps ratio, pain, KOOS-ADL, symptoms, and QOL** | **Dynamometry, KOOS** | **48-96 Weeks** | **Experimental group: 14 males, 11 females; conventional rehabilitation group: 14 males, 12 females** | **Experimental group: 27.7 (5.7), conventional rehabilitation group: 27.0 (6.4)** | **Experimental group: 78.7 (15.8), conventional rehabilitation group: 77.3 (14.7)** | **Experimental group: 175.3 (9.3), conventional rehabilitation group: 177.2 (8.9)** | **Experimental group: 25.6 (4.5), conventional rehabilitation group: 24.5 (3.4)** | **Hamstring autograft** |
| **Maroufi et al. 2023** | **RCT** | **ACLR** | **6 Weeks** | **Range of motion, pain, ACL-QOL, Timed Up and Go test (TUG), stair climbing test (SCT), 6-minute walk test (6MWT), tight abductor, adductor, extensor, flexor, external rotator, hamstring, and quadriceps strength** | **Goniometer, VAS, 36-item short form, isokinetic** | **1 Weeks** | **Experimental group: 22 males, 2 females; conventional rehabilitation group: 22 males, 2 females** | **Experimental group: 62.42 ± 3.59, conventional rehabilitation group:62.42±3.59** | **Experimental group: 72.29 ± 5.19, conventional rehabilitation group: 72.29±5.19** | **Experimental group: 163 ± 14.24, conventional rehabilitation group: 163 ± 14.24** | **Experimental group: 32±3.41, conventional rehabilitation group: 32±3.41** | **Hamstring autograft** |
| **Stojanovic et al. 2023** | **RCT** | **ACLR** | **6 Weeks** | **Strength (limb symmetry index (LSI)), single-leg hop, triple-leg hop, countermovement jump** | **Isokinetic, hop tests, LSI** | **20-24 Weeks** | **Experimental group: 4 females, 7 males; conventional rehabilitation group: 4 females, 7 males** | **Experimental group: 21.8 ± 4.6, conventional rehabilitation group: 19.1 ± 2.1** | **Experimental group: 82.7 ± 16.6, conventional rehabilitation group: 76.6 ± 16.5** | **Experimental group: 185.4 ± 12.2, conventional rehabilitation group: 182.5 ± 10.2** | **N/A** | **Hamstring autograft** |
| **Kasmi et al. 2023** | **RCT** | **ACLR** | **6 Weeks** | **Psychological measures, functional knee assessment, and knee flexor and extensor isokinetic muscle strength (limb symmetry index), peak torque (PT), total work, hamstring-quadriceps ratio peak torque, and ratio of total work at different angular velocities 90, 180, 240°/s)** | **TSK-CF, knee function questionnaire, isokinetic, KOOS, IKDC** | **14 Weeks** | **Experimental group: 10 males; conventional rehabilitation group: 10 males** | **Experimental group: 20.30 ± 2.83, conventional rehabilitation group: 20.4 ± 3.34** | **Experimental group: 78.00 ± 8.12, conventional rehabilitation group: 75.50 ± 5.23** | **Experimental group: 180.40 ± 10.12, conventional rehabilitation group: 179.60 ± 4.74** | **Experimental group: 21.59 ± 1.29, conventional rehabilitation group: 20.95 ± 1.09** | **Hamstring autograft** |
| **Wang et al. 2023** | **RCT** | **ACLR** | **4 Weeks** | **Knee flexion and extension isokinetic muscle strength** | **Isokinetic** | **4 Weeks** | **Experimental group: 7 males, 14 females; conventional rehabilitation group: 7 males, 13 females** | **Experimental group: 21.6 ± 3.2, conventional rehabilitation group: 22.2 ± 2.8** | **Experimental group: 69.5 ± 11.3, conventional rehabilitation group: 70.0 ± 12.5** | **Experimental group: 176.0 ± 8.2, conventional rehabilitation group: 175.6 ± 7.6** | **N/A** | **Hamstring autograft** |
| **Moubarak et al. 2022** | **RCT** | **ACLR** | **12 Weeks** | **Pain, range of motion, single-leg hop** | **VAS, goniometer, hoping tests** | **1 Weaks** | **Experimental group: 25 males; conventional rehabilitation group: 25 males** | **Experimental group: 30.0 ± 8.7, conventional rehabilitation group: 29.5 ± 7.6** | **Experimental group: 71.3 ± 4.9, conventional rehabilitation group: 69.3 ± 5.4** | **Experimental group: 169.1 ± 6.1, conventional rehabilitation group: 169.92 ± 11.2** | **Experimental group: 24.7 ± 2.7, conventional rehabilitation group: 23.8 ± 2.86** | **Hamstring autograft** |
| **Smith et al. 2022** | **RCT** | **ACLR** | **8 Weeks** | **Maximal voluntary isometric knee extension and flexion torque at 60°, quadriceps and hamstrings strength** | **Dynamometer** | **6 Weeks** | **Experimental group: 6 females, 2 males; conventional rehabilitation group: 4 females, 4 males** | **Experimental group: 22.1 ± 4.5, conventional rehabilitation group: 20.2 ± 5.3** | **Experimental group: 70.3 ± 12.2, conventional rehabilitation group: 70.2 ± 13.3** | **Experimental group: 172.3 ± 7.9, conventional rehabilitation group: 169.8 ± 9.0** | **N/A** | **10 Patient bone patellar tendon bone graft, 6 hamstring autograft** |
| **Minshull et al. 2021** | **RCT** | **ACLR** | **8 Weeks** | **Quadriceps peak force, hamstrings peak force, rate of force development, quadriceps and hamstring ratio, single leg hop test** | **Isokinetic, hop test** | **2 Weeks** | **Experimental group: 19 males; conventional rehabilitation group: 15 males** | **Experimental group: 33.3 (10.0), conventional rehabilitation group: 30.4 (9.4)** | **Experimental group: 80.6 (11.8), conventional rehabilitation group: 82.6 (18.2)** | **Experimental group: 174.8 (9.4), conventional rehabilitation group: 172.1 (9.5)** | **N/A** | **Hamstring autograft** |
| **Milandi et al. 2021** | **RCT** | **ACLR** | **8 Weeks** | **Knee flexion angle, moment. Hip angle, moment. Knee valgus angle, knee abduction moment, knee tibial rotation, concentric and eccentric quadriceps and hamstrings at 60° and 120°** | **Isokinetic** | **10-16 Weeks** | **Experimental group: 12 males; conventional rehabilitation group: 10 males** | **Experimental group: 25.86 ± 6.4, conventional rehabilitation group: 25.2 ± 6 6.0** | **Experimental group: 74.7 ± 13.2, conventional rehabilitation group: 79.4 ± 9.2** | **Experimental group: 175.6 ± 0.06, conventional rehabilitation group: 179 ± 0.06** | **N/A** | **Hamstring autograft** |
| **Kasmi et al. 2021** | **RCT** | **ACLR** | **6 Weeks** | **Single leg hop, 6-meter walk test, triple jump cross, triple hop test** | **Lysholm knee scale, return to sport index, hop tests** | **14 Weeks** | **Experimental group: 10 females; conventional rehabilitation group: 10 females** | **Experimental group: 20.3 ± 3.1, conventional rehabilitation group: 20.3 ± 3.3** | **Experimental group: 67.1 ± 8.0, conventional rehabilitation group: 64.2 ± 7.0** | **Experimental group: 170.7 ± 8.0, conventional rehabilitation group: 171.6 ± 9.6** | **Experimental group: 19.6 ± 1.5, conventional rehabilitation group: 18.6 ± 1.6** | **Hamstring autograft** |
| **Bette et al. 2021** | **RCT** | **ACLR** | **12 Weeks** | **84 Marker mRNAs** | **Biopsies** | **12 Weeks** | **Experimental group: 18 males, conventional rehabilitation group: 13 males** | **Experimental group: 24 ± 4, conventional rehabilitation group: 27 ± 5** | **Experimental group: 86.5 ± 16.4, conventional rehabilitation group: 80.1 ± 9.3** | **Experimental group: 182 ± 7, conventional rehabilitation group: 179 ± 6** | **N/A** | **19 Quadriceps and 12 hamstring autograft** |
| **Vidmar et al. 2020** | **RCT** | **ACLR** | **6 Weeks** | **Quadriceps muscle mass, Peak quadriceps strength, self-aware functionality, single leg hop test** | **Magnetic resonance imaging, isokinetic dynamometry, Lysholm scale, hoping tests** | **24 Weeks** | **Experimental group: 15 males; conventional rehabilitation group: 15 females** | **Experimental group: 26.9 ± 5.8, conventional rehabilitation group: 24.3 ± 4.6** | **Experimental group: 78.2 ± 8.7, conventional rehabilitation group: 78.7 ± 6.0** | **Experimental group: 178.5 ± 8.6, conventional rehabilitation group: 178.4 ± 8.9** | **Experimental group: 23.4 ± 1.0, conventional rehabilitation group: 23.7 ± 1.2** | **Hamstring autograft** |
| **Nadia et al. 2018** | **RCT** | **ACLR** | **6 Weeks** | **Effusion, hip extensor force, knee extensors torque, ankle plantar flexors force, Western Ontario and McMaster Universities Osteoarthritis Index (WOMAC), Time Up and Go test** | **Effusion grading scale, handheld dynamometer, chronometer** | **1 Weaks** | **Experimental group: 15 males; conventional rehabilitation group: 15 males** | **Experimental group: 24.61 ± 5.78, conventional rehabilitation group: 26.38 ± 5.47** | **Experimental group: 85.61 ± 11.02, conventional rehabilitation group: 81.07 ± 11.09** | **Experimental group: 177.15 ± 6.33, conventional rehabilitation group: 174.07 ± 6.66** | **Experimental group: 27.23 ± 2.62, conventional rehabilitation group: 26.66 ± 2.35** | **Hamstring graft or patellar tendon graft** |
| **Bette et al. 2018** | **RCT** | **ACLR** | **12 Weeks** | **Muscle cross-sectional area (MCSA), Fiber cross-sectional areas (FCSA), satellite cell, myogenic cell activation, type of autograft in angular velocities at 60°/s and 180°/s** | **Magnetic Resonance Imaging, isokinetic** | **12 Weeks** | **Experimental group: 21 males; conventional rehabilitation group: 16 males** | **Experimental group: 24 ± 4, conventional rehabilitation group: 26 ± 5** | **Experimental group: 87.0 ± 15.7, conventional rehabilitation group: 79.0 ± 12.7** | **Experimental group: 182.3 ± 6.8, conventional rehabilitation group:178.8 ± 5.9** | **N/A** | **Quadriceps and hamstring autograft** |
| **Bell et al. 2016** | **RCT** | **ACLR** | **24 Weeks** | **Knee extension, knee flexion, hip abduction, hip extension, hip internal rotation, hip external rotation strength, knee evaluation, activity level, and strength LSI** | **Isokinetic, IKDC, Marx Activity Scale, knee extension strength test** | **30.9 ± 17.6 months** | **Experimental group: 31 females, 6 males; conventional rehabilitation group: 17 females, 1 male** | **Experimental group: 19.1 ± 1.7, conventional rehabilitation group: 18.7 ± 1.6** | **Experimental group: 70.5 ± 13.3, conventional rehabilitation group: 66.6 ± 8.2** | **Experimental group: 169.7 ± 6.8, conventional rehabilitation group: 167.2 ± 7.2** | **N/A** | **26 Patient bone patellar tendon bone graft, 29 hamstring autograft** |
| **Kinikli et al. 2014** | **RCT** | **ACLR** | **12 Weeks** | **Flexion and extension total work, vertical jump test, single hop test, peak torque of the knee extensors and flexors at 60° and 180°, functional performance (the vertical jump test, single hop, ACL-QOL)** | **Isokinetic, hop tests, Lysholm knee scale, functional questionnaire** | **3 Weeks** | **Experimental group: 15 males, 1 female; conventional rehabilitation group: 15 males, 2 females** | **Experimental group: 33.87±8.19, conventional rehabilitation group: 32.64±8.21** | **N/A** | **N/A** | **Experimental group: 24.50±2.36, conventional rehabilitation group: 24.52±0.94** | **Hamstring autograft** |
| **Garrison et al. 2014** | **RCT** | **ACLR** | **12 Weeks** | **Pain, function, range of motion** | **VAS, IKDC, goniometer** | **1 Weaks** | **Experimental group: 12 males, 9 females; conventional rehabilitation group: 10 males, 12 females** | **Experimental group: 14-40, conventional rehabilitation group: 14-40** | **N/A** | **N/A** | **N/A** | **38 Patients with Patellar tendon and 5 hamstring** |
| **Gerber et al. 2009** | **RCT** | **ACLR** | **12 Weeks** | **Quadriceps femoris and gluteus maximus muscle volume, single leg hop, and quadriceps femoris muscle strength and hamstring muscle strength** | **Magnetic resonance imaging, hop tests, isokinetic, ADL scale, Lysholm knee scale** | **3 Weeks** | **Experimental group: 17 males and females; conventional rehabilitation group: 15 males and females** | **Experimental group: 29.36 ± 8.6, conventional rehabilitation group: 29.36 ± 9.7** | **Experimental group: 78.06 ± 17.0, conventional rehabilitation group: 76.56 ± 12.4** | **Experimental group: 176.66 ± 9.3, conventional rehabilitation group: 174.76 ± 10.3** | **N/A** | **12 Patient bone patellar tendon bone graft, 20 hamstring autograft** |
| **Shaw et al. 2005** | **RCT** | **ACLR** | **24 Weeks** | **Active, passive knee flexion and extension ROM, inferior knee circumference (LSI%), pain, single hop test, triple hop test, Cincinnati Knee Rating System (CKRS) symptoms, patient grade, sports activity score, ADL function score, sports function score, concentric and eccentric quadriceps at 60° (LSI)** | **Goniometer, VAS, hop tests, CKRS scale, isokinetic** | **2 Weeks** | **Experimental group: 14 female, 34 male; conventional rehabilitation group: 14 female, 41 male** | **Experimental group: 28.8 ± 9.3, conventional rehabilitation group: 28.4 ± 8.1** | **Experimental group: 80.7 ± 12.4, conventional rehabilitation group: 76.5 ± 12.6** | **Experimental group: 177.9 ± 7.4, conventional rehabilitation group: 175.0 ± 9.7** | **N/A** | **63 Patients patella tendon graft, 40 hamstring graft** |
