# Supplementary material for: Strengthening exercises improve knee muscle strength and performance but not pain in ACL‐reconstructed individuals: A systematic review and meta‐analysis of randomised controlled trials
Source: J Exp Orthop. 2025 Dec 17;12(4):e70576. doi: 10.1002/jeo2.70576 (PMC12709656; doi:10.1002/jeo2.70576)
Supplement: Supplementary file 3 — Table 3. Definitions of modified level of evidence by Jalili Bafrouei. [file JEO2-12-e70576-s005.docx]

Table 3. Definitions of modified level of evidence by Jalili Bafrouei.

| Level of evidence | Description |
| --- | --- |
| Strong evidence | Pooled results from three or more studies, including a minimum of two high-quality studies, such as the PEDro scale for RCTs, the Newcastle-Ottawa scale (NOS) for non-RCTs, and which are statistically homogenous (p>0.05), or may be associated with statistically significant or non-significant pooled results. An I² cut-off to aid interpretability (I² < 50% = low, 50–74% = medium, > 75% = high). |
| Moderate evidence | Statistically significant pooled results from multiple studies, including at least one high-quality study, which are statistically heterogeneous (p<0.05); or from multiple low- or moderate-quality studies which are statistically homogenous (p>0.05); or statistically insignificant pooled results from multiple studies, including at least one high-quality study, which are statistically homogenous (p>0.05). An I² cut-off to aid interpretability (I² < 50% = low, 50–74% = medium, > 75% = high). |
| Limited evidence | Results from multiple low- or moderate-quality studies, which are statistically heterogeneous (p<0.05), or from one high-quality study. An I² cut-off to aid interpretability (I² < 50% = low, 50–74% = medium, > 75% = high). |
| Very limited evidence | Results from one low- or moderate-quality study. |
| Conflicting evidence | Pooled results that are insignificant and from multiple studies, regardless of quality, which are statistically heterogeneous (p<0.05, i.e., inconsistent). |
