# Supplementary material for: Strengthening exercises improve knee muscle strength and performance but not pain in ACL‐reconstructed individuals: A systematic review and meta‐analysis of randomised controlled trials
Source: J Exp Orthop. 2025 Dec 17;12(4):e70576. doi: 10.1002/jeo2.70576 (PMC12709656; doi:10.1002/jeo2.70576)
Supplement: Supplementary file 4 — Table 4. The results of the PEDro quality assessment scale in RCTs. [file JEO2-12-e70576-s002.docx]

Table 4. The results of the PEDro quality assessment scale in RCTs.

| Study | Random allocation | Concealed allocation | Baseline comparability | Blinding of participants | Blinding of therapists | Blinding of assessors | Adequate of follow-up (>85%) | Intention-to-treat analysis | Between-group statistical comparisons | Reporting of point measures and measures of variability | Overall |
| --- | --- | --- | --- | --- | --- | --- | --- | --- | --- | --- | --- |
| Bregenhof et al. 2023 | 1 | 1 | 1 | 1 | 0 | 1 | 1 | 1 | 1 | 1 | 9 |
| Maroufi et al. 2023 | 1 | 0 | 1 | 0 | 0 | 0 | 1 | 1 | 1 | 1 | 6 |
| Stojanvic et al. 2023 | 1 | 0 | 1 | 0 | 0 | 1 | 1 | 1 | 1 | 1 | 7 |
| Kasmi et al. 2023 | 1 | 1 | 1 | 0 | 0 | 0 | 1 | 1 | 1 | 1 | 7 |
| Wang et al. 2023 | 1 | 0 | 1 | 0 | 0 | 0 | 1 | 1 | 1 | 1 | 6 |
| Moubarak et al. 2022 | 1 | 1 | 1 | 1 | 1 | 1 | 1 | 1 | 1 | 1 | 10 |
| Smith et al. 2022 | 1 | 0 | 1 | 0 | 0 | 0 | 1 | 1 | 1 | 1 | 6 |
| Minshull et al. 2021 | 1 | 0 | 1 | 0 | 0 | 0 | 1 | 1 | 1 | 1 | 6 |
| Milandi et al. 2021 | 1 | 1 | 1 | 1 | 1 | 0 | 1 | 1 | 1 | 1 | 9 |
| Kasmi et al. 2021 | 1 | 1 | 1 | 0 | 0 | 0 | 1 | 1 | 1 | 1 | 7 |
| Bette et al. 2021 | 1 | 0 | 1 | 0 | 0 | 0 | 1 | 1 | 1 | 1 | 6 |
| Vidmar et al. 2020 | 1 | 0 | 1 | 0 | 0 | 1 | 1 | 1 | 1 | 1 | 7 |
| Nadia et al. 2018 | 1 | 0 | 1 | 0 | 0 | 0 | 1 | 1 | 1 | 1 | 6 |
| Bette et al. 2018 | 1 | 0 | 1 | 1 | 1 | 1 | 1 | 1 | 1 | 1 | 9 |
| Bell et al. 2016 | 1 | 0 | 1 | 0 | 0 | 0 | 1 | 1 | 1 | 1 | 6 |
| Kinikli et al. 2014 | 1 | 0 | 1 | 1 | 0 | 0 | 1 | 1 | 1 | 1 | 7 |
| Garrison et al. 2014 | 1 | 0 | 0 | 0 | 0 | 0 | 1 | 1 | 1 | 1 | 5 |
| Gerber et al. 2009 | 1 | 0 | 1 | 0 | 0 | 0 | 0 | 1 | 1 | 1 | 5 |
| Shaw et al. 2005 | 1 | 0 | 1 | 1 | 1 | 1 | 1 | 1 | 1 | 1 | 9 |
