# Supplementary material for: Strengthening exercises improve knee muscle strength and performance but not pain in ACL‐reconstructed individuals: A systematic review and meta‐analysis of randomised controlled trials
Source: J Exp Orthop. 2025 Dec 17;12(4):e70576. doi: 10.1002/jeo2.70576 (PMC12709656; doi:10.1002/jeo2.70576)
Supplement: Supplementary file 5 — Table 5. Risk of bias in RCTs. [file JEO2-12-e70576-s007.docx]

Table 5. Risk of bias in RCTs

| Study | Randomization process | Deviations from intended intervention | Missing outcome data | Measurement of the outcome | Selection of the reported result | Overall |  |  |
| --- | --- | --- | --- | --- | --- | --- | --- | --- |
| Bregenhof et al. 2023 | + | + | + | + | + | + |  |  |
| Maroufi et al. 2023 | + | ? | + | + | + | ? |  |  |
| Stojanvic et al. 2023 | + | ? | + | + | + | ? |  |  |
| Kasmi et al. 2023 | + | ? | + | + | + | ? |  |  |
| Wang et al. 2023 | + | ? | + | + | + | ? |  |  |
| Moubarak et al. 2022 | + | ? | + | ? | + | ? |  |  |
| Smith et al. 2022 | + | ? | + | + | + | ? |  |  |
| Minshull et al. 2021 | + | ? | + | + | + | ? |  |  |
| Milandi et al. 2021 | + | ? | + | + | + | ? |  |  |
| Kasmi et al. 2021 | + | ? | + | ? | + | ? |  |  |
| Bette et al. 2021 | + | ? | + | + | + | ? |  |  |
| Vidmar et al. 2020 | + | ? | + | + | + | ? |  |  |
| Gerber et al. 2009 | + | + | ? | + | + | ? |  |  |
| Nadia et al. 2018 | + | ? | + | + | + | ? |  |  |
| Bette et al. 2018 | + | ? | + | + | + | ? |  |  |
| Bell et al. 2016 | + | ? | + | + | + | ? |  |  |
| Kinikli et al. 2014 | + | ? | + | + | + | ? |  |  |
| Garrison et al. 2014 | + | - | + | ? | + | - |  |  |
| Shaw et al. 2005 | + | - | + | + | + | - |  |  |
|  |  |  |  |  |  |  | + | Low risk |
|  |  |  |  |  |  |  | ? | Some concerns |
|  |  |  |  |  |  |  | - | High risk |
