# Supplementary material for: Strengthening exercises improve knee muscle strength and performance but not pain in ACL‐reconstructed individuals: A systematic review and meta‐analysis of randomised controlled trials
Source: J Exp Orthop. 2025 Dec 17;12(4):e70576. doi: 10.1002/jeo2.70576 (PMC12709656; doi:10.1002/jeo2.70576)
Supplement: Supplementary file 6 [file JEO2-12-e70576-s006.docx]

Risk of publication bias for quadriceps muscle strength in the SE group compared to the conventional rehabilitation group.

Risk of publication bias for the single-leg hop test in the SE group compared to the conventional rehabilitation group.

Risk of publication bias for the THT test in the SE group compared to the conventional rehabilitation group.

Risk of publication bias for the THT test in the SE group compared to the conventional rehabilitation group
