# Supplementary material for: Strengthening exercises improve knee muscle strength and performance but not pain in ACL‐reconstructed individuals: A systematic review and meta‐analysis of randomised controlled trials
Source: J Exp Orthop. 2025 Dec 17;12(4):e70576. doi: 10.1002/jeo2.70576 (PMC12709656; doi:10.1002/jeo2.70576)
Supplement: Supplementary file 7 [file JEO2-12-e70576-s003.docx]

Meta-analysis comparing hip abduction muscle strength (SMD: 0.35, SE: 0.20) between group B (the SE group, N=61) and group A (the conventional rehabilitation group, N=42).

.

Meta-analysis comparing the isokinetic concentric quadriceps muscle at 60°/s (SMD: 0.06, SE: 0.17) between group B (the SE group, N=60) and group A (the conventional rehabilitation group, N=65).

Meta-analysis comparing the isokinetic eccentric quadriceps muscle at 60°/s (SMD: 0.07, SE: 0.17) between group B (the SE group, N=60) and group A (the conventional rehabilitation group, N=65).

Meta-analysis comparing the TUG (SMD: 0.83, SE: 0.35) between group B (the SE group, N=39) and group A (the conventional rehabilitation group, N=39).

Meta-analysis comparing the 6-MWT (SMD: 1.05, SE: 0.62) between group B (the SE group, N=34) and group A (the conventional rehabilitation group, N=34).

.

Meta-analysis comparing the QOL questionnaire (SMD: 0.62, SE: 0.43) between group B (the SE group, N=65) and group A (the conventional rehabilitation group, N=67).

Meta-analysis comparing the THT (SMD: 1.41, SE: 0.99) between group B (the SE group, N=69) and group A (the conventional rehabilitation group, N=66).

Meta-analysis comparing the IKDC (SMD: 1.78, SE: 0.95) between group B (the SE group, N=49) and group A (the conventional rehabilitation group, N=69).
