# Supplementary material for: Strengthening exercises improve knee muscle strength and performance but not pain in ACL‐reconstructed individuals: A systematic review and meta‐analysis of randomised controlled trials
Source: J Exp Orthop. 2025 Dec 17;12(4):e70576. doi: 10.1002/jeo2.70576 (PMC12709656; doi:10.1002/jeo2.70576)
Supplement: Supplementary file 8 [file JEO2-12-e70576-s004.docx]

Hamstrings muscle strength*

Quadriceps muscle strength*

**Knee Strength**

Hip external rotation muscle strength*

↔Hip abduction muscle strength

↔Eccentric quadriceps muscle at 60°/s

↔Concentric quadriceps muscle at 60°/s

SLHT*

ADL questionnaire*

## Performance

Lysholm scale*

ROM in knee extension*

TUG test*

↔6-MWT

↔QOL questionnaire

↔IKDC scale

↔THT

**Pain**

↔VAS

Increased, Decreased, ↔Lack of significant, *Significant

Strong evidence, Moderate evidence, Conflicting evidence

*A graphical abstract of important SE factors based on the meta-analysis results*
